# Feasibility of Unbiased RNA Profiling of Colorectal Tumors: A Proof of Principle

**DOI:** 10.1371/journal.pone.0159522

**Published:** 2016-07-21

**Authors:** Vardit Moshayoff, Ouriel Faktor, Luigi Laghi, Giuseppe Celesti, Tamar Peretz, Dan Keret, Dana Cohen, Eran Israeli

**Affiliations:** 1 Bio-Marcare Technologies, BioPark, Jerusalem, Israel; 2 Humanitas Clinical and Research Center, Rozzano, Milan, Italy; 3 Sharett Institute of Oncology, Hadassah-Hebrew University Medical Center, Jerusalem, Israel; 4 Gastroenterology and Hepatology Department, Clalit Health Services, Jerusalem, Israel; 5 Institute of Gastroenterology and Liver Diseases, Hadassah-Hebrew University Medical Center, Jerusalem, Israel; Sapporo Medical University, JAPAN

## Abstract

Despite recent advances in molecular profiling of colorectal cancer (CRC), as of yet this has not translated into an unbiased molecular liquid biopsy profile which can accurately screen for early CRC. In this study we depict the profile of early stage CRC as well as for advanced adenomas (AA) by combination of current molecular knowledge with microarray technology, using efficient circulating free plasma RNA purification from blood and RNA amplification technologies. We joined literature search with Affymetrix gene chip experimental procedure to draw the circulating free plasma RNA profile of colorectal cancer disease reflected in blood. The RNA panel was tested by two datasets comparing patients with CRC with healthy subjects and patients with AA to healthy subjects. For the CRC patient cohort (28 CRC cases vs. 41 healthy controls), the ROC analysis of the selected biomarker panel generated a sensitivity of 75% and a specificity of 93% for the detection of CRC using 8-gene classification model. For the AA patient cohort (28 subjects vs. 46 healthy controls), a sensitivity of 60% and a specificity of 87% were calculated using a 2-gene classification model. We have identified a panel of 8 plasma RNA markers as a preliminary panel for CRC detection and subset markers suitable for AA detection. Subjected to extensive clinical validation we suggest that this panel represents a feasible approach and a potential strategy for noninvasive early diagnosis, as a first-line screening test for asymptomatic, average-risk population before colonoscopy.

## Introduction

Colorectal cancer (CRC) is the third most common cancer and the fourth cause of cancer death in the world. CRC is amenable to both early diagnosis and prevention [1]. This has been made possible through the availability of fecal occult blood testing and colonoscopy. Both have been tested by an array of clinical and population studies, showing that they result in reduced CRC mortality, and morbidity [2–4]. These tests differ regarding invasiveness, screening adhesion rates, and costs with variable success end-points.[5, 6]

In parallel, molecular profiling of CRC also produced relevant clinical implications, such as molecular diagnosis of inherited predispositions.[7] This has allowed at the population level to introduce the concept of molecular screening, and development of tailored preventive strategies.[8] As a consequence of increasing molecular knowledge on tumor development, innovative technologies have been applied to historical screening tools for fecal occult blood testing. This has recently led to development of a hybrid device for CRC diagnosis and prevention (Cologuard).[9] Additional developments in this field include diagnosis of CRC, and advanced adenomas (AA) by nucleic-acid amplification technologies applied to blood samples. The feasibility of such an approach is testified by a large number of studies.[10–12] The identification of a circulating RNA fingerprint specific for CRC could potentially perform better than the search for tumor specific DNA alterations. This is because expression profiles expand beyond the mutational spectrum in depicting molecular changes associated with neoplasia development and progression.[13–16] Although there are a few examples of the feasibility of detection of CRC and AA by profiling circulating RNAs, this has not translated into an unbiased profile of CRC.[17–21] We aimed to depict the profile of CRC by combination of current molecular knowledge with microarray technology, so as to maximize the information available through bioinformatics and experimental data. We employed a strategy for fishing information from the literature, and merging this data with that obtained by microarray analysis of circulating RNAs from patients with CRC. This enabled us to derive a reliable RNA profile with potential diagnostic implications. Our results first demonstrate that molecular diagnosis of CRC is feasible from a blood sample in the average risk population; Secondly, they enable to introduce the notion of unbiased molecular RNA profiling as a tool for early CRC diagnosis.

## Materials and Methods

We joined literature search with Affymetrix gene chip experimental procedure to draw the RNA profile of CRC reflected in blood. Methodological approach and technical details are presented in Tables 1 and 2 and are detailed for each step below.

**Table 1:** Workflow from gene screening, through gene selection, to experimental identification of a disease predictor.

	Gene Discovery Steps			
**❶**	**Group A**		**Group B**	
	**Bioinformatics Based on Literature Survey 500 Peer Review Papers**		**Plasma Gene Expression Analysis (Affymetrix Arrays)**	
**❷**	**Gene Lists Extracted From 3 Selected Peer Review Papers**			**Expressed Gene List**
**❸**	**List 1 Tissue Arrays** 28 Advanced adenoma 3 Control **1,462 genes** [22, 23]	**List 2 Plasma Array** 12 CRC, 8 Control **4,828 genes**[24]	**List 3 PBMCs Arrays** 100 CRC, 100 Control **2,272 genes**[25]	**Affymetrix Arrays** 4 Advanced adenoma 3 CRC, 3 Control **6,577 genes**
**❹**	**Selection of Common Genes Between Affymetrix Arrays and Different Gene Lists** (Table 2)			
**❺**	**Selection of 72 Genes**			
**❻**	**Screening for circulating free plasma RNA Expression by qPCR using 15–20 plasma samples for each gene** (Advanced adenoma, CRC, Control)			
❼		**Selection of 17 Genes**		
**❽**	**Testing for Differential circulating free plasma RNA Expression by qPCR using 50–100 plasma samples for each gene** (Advanced adenoma, CRC, Control)			
**❾**	**Final Selected Gene Set of 8 Genes**			
**❿**	**Training Set: Study Cohort of 144 Plasma Samples** 48 Advanced adenoma, 36 CRC, 60 Control			
**⓫**	**Statistical Analyses**			
**⓬**	**Disease Predictor Algorithms**			

Affymetrix arrays data were deposited in Gene Expression Omnibus, accession number: GSE83353

**Table 2:** Numbers of common genes between Affymetrix arrays and gene lists and number of genes used for the different steps of qPCR selections.

No. of Expressed Genes in Affymetrix Arrays: 6,577				
	Bioinformatics	1^st^ qPCR selection	2^nd^ qPCR selection	Statistical Analyses
Gene list *c*ombinations	No. of common genes between Affymetrix Arrays and gene lists	No. of selected genes	No. of selected genes	No. of gene in final gene set
**List 1**	1,463	14	7	2
**List 2**	522	13	4	2
**List 3**	98	0	0	0
**List 1+List 2**	515	16	1	0
**List 1+List 3**	91	2	0	0
**List 2+List 3**	7	2	2	2
**List1+List 2+List 3**	35	19	1	1
**List 4**		6	2	1
	**Total**	72	17	8

### Literature search

Scientific publications were searched for gene expression data related to CRC, using a set of key-words representing common cancer and molecular biology terms. Three out of about 500 publications identified by the search were chosen and the supplemented gene expression data was retrieved.

### Study population

Overall recruitment for the clinical multicenter study included 850 eligible subjects who provided written informed consent. Samples were subdivided according to the following groups: 37 subjects with CRC; 109 subjects with AA; 132 subjects with non-advanced adenomatous polyps; 29 subjects with non-adenomatous polyps and 543 subjects with no colonoscopic findings. The study took place at Gastroenterology Departments of Hadassah Medical Center, Israel; Humanitas Clinical and Research Center, Italy and Clalit Health Services, Israel. The study was approved by the Helsinki Committee of Hadassah- Hebrew University Medical Center, The independent Ethical Committee of Humanitas Clinical and Research Center, and the Helsinki Committee of Clalit Health Services.

The group of patients diagnosed with CRC provided a 10 ml whole-blood sample drawn into vacutainer collection tubes, prior to surgery. Other study groups included subjects who were pre-scheduled for colonoscopy. Specific high-risk groups were excluded, including those with previous CRC or adenomas, a family history indicating increased risk for the disease. On the day of colonoscopy s 10 ml whole blood was drawn into a vacutainer collection tube. Colonoscopy procedures, including polypectomy and biopsy, were performed by board certified endoscopists using screening standards and site specific standards for sedation, monitoring, imaging and equipment. Histopathology, diagnostic procedures, and staging of biopsy and surgical specimens used routine procedures. The groups of non-advanced adenomatous polyps and non-adenomatous polyps were not included in this study.

For the development and calibration of circulating free plasma RNA purification method and for the discovery of circulating free plasma biomarkers in plasma, a group of about 500 blood samples was used.

For the analysis of the clinical performance of the selected gene profile a training-set cohort of 144 subjects was constructed out of the pool of samples. The patient cohort is depicted in Table 3 (48 AA, 36 with CRC, and 60 Healthy subjects).

**Table 3:** Clinical and histological data of study cohort.

	Healthy N = 60 (%)	Advanced Adenoma N = 48 (%)	CRC N = 36 (%)
**Age**			
<50	13 (22)	2 (4)	5 (14)
50-<60	19 (32)	12 (26)	6 (16)
60-<70	18 (30)	16 (33)	10 (28)
70-<80	9 (14)	16 (33)	14 (39)
80+	1 (2)	2 (4)	1 (3)
**Gender**			
Male	33 (55)	30 (63)	19 (53)
Female	27 (45)	18 (37)	17 (47)
**Location**			
Rectum		4 (8)	11 (31)
Left		17 (36)	13 (36)
Right		25 (52)	12 (33)
UK		2 (4)	
**Size**			
<1 cm		12 (26)	
> = 1cm		36 (74)	
> = 3cm			
**Villous component**			
+		29 (60)	
-		19 (40)	
**TD**			
Well			5 (14)
Moderate			21 (58)
Poor			5 (14)
UK			5 (14)
**Stage**			
I			5 (14)
II			19 (53)
III			11 (30)
IV			0 (0)
UK			1 (3)

### RNA specimen preparation for gene expression profiling using Affymetrix gene arrays

Total RNA was purified according to the following protocol: eight micro tubes containing the TRIzol-plasma mixture of the same individual were thawed on ice and 15 microgram of linear acrylamide (K548, Amresco) and 200 μl of chloroform (catalog no. 4443–06, J.T. Baker) were added per each 1 ml of Trizol were mixed vigorously. After incubated for 10 minutes at room temperature, the mixture was centrifuged for 15 minutes at 14000 rpm at 4°C. The aqueous phase was transferred to a new tube and mixed vigorously with equal volume of chloroform, incubated for 3 minutes at room temperature and centrifuged for 15 minutes at 14,000 rpm at 4°C. Following the centrifugation, the upper phase was transferred to a new tube and further RNA purification steps were performed as above described for RNA specimen preparation for qPCR.

### Affymetrix Array Test Procedure

This procedure requires the use of the GeneChip^®^ Hybridization, Wash and Stain Kit (catalog no. 900720, Affymetrix) and the GeneChip human 1.0 ST Arrays (catalog no. 901085, Affymetrix). Hybridization cocktail was prepared by mixing the following materials: 25 microliter of fragmented, biotin-labeled and amplified cDNA, 1.9 microliter control oligonucleotide B2 (3 nM), 5.5 microliter of 20X Eukaryotic hybridization controls (bioB, bioC, bioD, cre), 55 microliter 2X Hybridization Buffer, 11 microliter 100% DMSO, 11.6 microliter water. The procedure was performed according to manufacturer instructions.

### cDNA synthesis and modifications

For testing gene expression levels by qPCR, 10 microliter of plasma RNA was used for each cDNA reaction. The Reverse Trascriptase reaction was performed with qScript buffer mix and RT enzyme (catalog no. 95047, Quanta) in a final reaction volume of 20 microliter. RT reaction conditions were incubation at 22°C for 5 minutes, 42°C for 30 minutes and 85°C for 5 minutes. The produced cDNA was stored at -20°C. For gene expression profiling using Affymetrix expression microarray, cDNA was synthesized using the Ovation PicoSL WTA System (catalog no. 3312, Nugen), according to kit's protocol. Subsequently, the cDNA was purified with MiniElute Reaction Cleanup kit (catalog no. 28204, Qiagen), according to kit protocol. Next, fragmentation and biotin labeling of the cDNA was done by Encore biotin module (catalog no. 4200, Nugen). The extent of fragmentation was monitored using Bioanalyzer 2100 Pico chip (Agilnet) using 1 μl of the reaction volume.

### RNA Specimen Preparation for qPCR

Ten ml of blood were collected (vacutainer, catalog no. 367525, BD) before surgery or before colonoscopy. Plasma was separated from blood cells by centrifugation and homogenized with TRIzol^®^ Reagent (catalog no. 15596–026, Invitrogen). Each volume of plasma was mixed with 3.5 volumes of TRIzol reagent. The mixture was divided into storage micro tubes and stored at -80°C.

For total RNA extraction four micro tubes containing the TRIzol-plasma mixture of the same individual were thawed on ice. To each 1 ml TRIzol-plasma mixture one microgram of MS2 RNA (catalog no. 10-165-948, Roche) and 200 micro liter of chloroform (catalog no. 4443–06, J.T. Baker) were added. The solution was mixed vigorously and incubated for 10 minutes at room-temperature. Subsequently, the mixture was centrifuged for 15 minutes at 14,000 rpm at 4°C. The aqueous phase was transferred to a new tube and mixed vigorously with equal volume of chloroform, incubated for 3 minutes at room-temperature and centrifuged for 15 minutes at 14,000 rpm at 4°C. Following centrifugation, the upper phase was transferred to a new micro tube mixed thoroughly with a total of 0.35 ml of RLT plus buffer RNeasy mini kit (catalog no. 74104, Qiagen) per each tube. This mixture was passed through the kit's gDNA eliminator mini-spin column as described by the manufacture protocol. The flow-through was collected in a new tube, and 1.5 times volume of 100% EtOH (catalog no. 830140370, Gadot, ISRAEL) per each tube was added. The solution was well mixed and incubated at -20°C over-night. The solution was thawed and 700 micro liter of the mixture was loaded on an RNeasy spin column and micro-centrifuged at 23°C, 14,000 RPM, for 30 seconds and flow-through was saved. The rest of the thawed solution was passed through the same RNeasy spin column as described above.

RNA purification process was completed by following the RNeasy mini kit protocol. The RNA-loaded RNeasy spin column were washed with 700 microliter of RW1 buffer, centrifuged at 23°C 14,000 RPM for 30 seconds and flow-through discarded. Additional two washes of spin column were done with 500 microliter of RPE buffer. Finally, RNA was eluted in 35 microliter of RNase-free water. For complete dissolution of the RNA, micro tubes containing the eluted RNA were incubated for 5 minutes at 65°C subsequently incubated on ice for 5 minutes and spun down. RNA quantity was measured using NanoDrop instrument (Thermo Scientific).

### qPCR method for circulating free plasma RNA quantification

For quantitation of gene expression levels the prepared cDNA was diluted 4 times in DDW molecular biology grade. In a typical qPCR reaction the PerfeCTa qPCR SuperMix (catalog no. 95065, Quanta) was used, together with 2 microliter of the diluted cDNA, forward and reverse primers set specific for each tested gene, hydrolysis probes in a final volume of 20 microliter. The qPCR was performed in a 96 well PCR plate, for 52 cycles according to Quanta's specified conditions, using ABI Prism 7900 system (Applied Biosystems). Two reference genes, human HPRT1 and human TFRC genes were used for normalization of gene expression levels of a tested gene. The primers and probe sequences used for PCR were as followed: hHPRT1 gene, Fw primer- TATGCTGAGGATTTGGAAAGG, Rev. primer—CATCTCCTTCATCACATCTCG (final concentration 300nM) Probe- FAM-TATGGACAGGACTGAACG-3'IABkFQ with addition of 4 LNAs (final concentration 200nM). hTFRC gene, Fw primer- TTGCATATTCTGGAATCCCA, Rev. primer- TCAGTTCCTTATAGGTGTCCATG (final concentration 500nM), Probe- FAM-TCTGTGTCCTCGCAAAAA-3'IABkFQ with addition of 5 LNAs (final concentration 250nM). For determining gene expression levels a Relative Quantity (RQ) value was calculated by the formula: RQ = 2^(-ΔCt), where the ΔCt is the difference between the Ct measured for a tested gene marker and the reference house-keeping genes. Calculations were performed by DataAssist v3.01 software (ABI).

### Statistical analysis

For all statistics analysis we used SPSS package, version 21 (IBM SPSS Statistics).

## Results

### Purification of circulating free RNA from plasma

Developing a methodology for quantifying RNA levels in plasma is challenging due to the scarce amount of RNA and its easily degradable state. Also for our specimens, examination of the quality and quantity of RNA prepared by the methods described here using Agilent bioanalyzer chip, revealed extremely low amounts, in the range of 500 nanograms, of highly degraded total RNA (Fig A in S1 File). In addition, no established tools are currently available for analyzing array’s gene expression for the discovery of plasma circulating free RNA.

Despite these unfavorable conditions we succeeded in developing an RNA purification procedure that provides reproducible qPCR results. To ensure a reliable normalization of gene expression by qPCR two out of 6 reference gene candidates were identified. The 6 candidate reference genes were: ACTB, HPRT, RPLPO, TBP, TFRC; beta-GUS And the expression was tested on 5 Healthy; 3 AA; 2 CRC and 3 IBS samples As can be seen in Fig B in S1 File, ACTB gene was expressed in early qPCR cycles (cycle 23–28), and beta-GUS gene was expressed in late qPCR cycles (34.5–39) and expression of TBP gene could not be detected in all samples. Therefore, we selected *HPRT1* and *TFRC* genes as reference genes, since they were the two stably expressed genes (5–6 cycles’ interval fluctuation) and were expressed at the same qPCR cycle range as target biomarker candidates that were screened (33–38 qPCR cycles). HPRT1 and TFRC genes were the most stably expressed genes as been calculated by DataAssist v3.1 software (Fig B in S1 File). Due to the low abundance of the targeted genes expected in plasma of AA subjects, a calibration of the primer-probe ratio was necessary. Calibration was performed by qPCR using cDNA dilution series and calibrated primer-probe ratio required to pass criteria of R^2^ > 0.96 and slope of -3.2x ± 10% as demonstrated in Fig C in S1 File.

### Molecular screening for colorectal tumor signature in blood by RNA expression levels

For optimal gene discovery, differential gene expression array results were combined with bioinformatics literature search and revealed a set of candidate genes.

Initially, to identify gene profile that is indicative to AA and early CRC vs. expression levels in plasma of healthy individuals a gene expression analysis using GeneChip human 1.0 ST Array of Affymetrix was performed. RNA samples from 4 AA cases and 3 CRC cases were randomly selected and processed on Affymetrix array, together with samples from 3 healthy subjects, for gene expression analysis. Initial analysis of these arrays showed low array signal level which prevented performing signals normalization and differentiation between the three tested groups. Instead, we set a signal frequency cut-off value (above level 4) assuming that gene expression above this cut off represents a true expression signal. Using this cut-off approached a probe level analysis of these arrays revealed the expression of 6,577 genes that shared a common presence in plasma of all subjects (Table 1, group B) with some differences in expression levels between healthy subjects and CRC or AA subjects.

The limited number of arrays used for this analysis and the lack of differentiation between the groups did not allow generating a disease specific RNA profile. To overcome this limitation we used bioinformatics literature search aimed to generate lists of genes the expression of which has been associated with early stage CRC.

The bioinformatics search yielded 4 gene lists (Table 1, group A). List 1 comprising 1462 genes was identified in plasma samples.[23, 24] List 2 of 4828 genes was identified in sessile serrated polyps or adenoma samples.[22] List 3 of 2,272 genes was identified in peripheral blood mononuclear cells samples.[25] Then, we matched the 3 bioinformatics gene lists with our list of genes expressed above signal cut-off experimentally generated by the GeneChip, using the Venny tool.[26] Genes shared between the Affymetrix chip arrays and the described lists were used for further gene selection (Table 2).

In addition, List 4 comprising of single arbitrarily selected candidate biomarkers that we included in the gene selection process. Genes of unknown function and pseudo-genes were excluded.

Further gene trimming was conducted by qPCR in order to establish an *In-Vitro* diagnostic assay. Due to the low circulating free plasma RNA copy number in plasma, primer to probe ratio optimization was required. For gene level normalization the two housekeeping genes HPRT1 and TFRC, were again used. The experimental qPCR test on samples was run in order to minimize the number of predictive biomarkers.

For the qPCR assay, 72 genes were chosen out the common genes lists (Table 2) based on inspection of the different probe signals for each gene and choosing probes with maximal signal intensity (Table 2). The 72 genes were tested with RNA prepared from plasma samples from the three clinical groups (15–20 samples for each gene). Out of the experimentally tested genes, 17 genes were identified as differentially expressed between control and disease samples, the rest of probes either failed to generate expressed product or did not meet the differential requirement (Table A in S1 File).

The next step was to increase the number of tested samples to 50–100 for each gene. Having increased the sample number allowed further trimming down to only 8 relevant genes (Table 2). To further study the role of each gene in this group a training-set was tested by qPCR. The clinical parameters of the study groups are depicted in Table 3.

Initial assessing of the clinical performance provided by each of the 8 genes showed that a single gene is not able to provide a desirable diagnostics. Therefore, the qPCR dataset for the 8 genes was subjected to statistical analyses, targeted to identify the best performing gene expression profile that can discriminate between non-cancerous and AA or CRC.

### Identifications of patient with colorectal tumors by analysis of specific RNA expression levels in plasma

Two datasets of qPCR ΔCt results were used for the analysis. The first set compared patient with CRC (n = 28) to healthy subjects (n = 41). The second set compared patient with AA (n = 38) to healthy subjects (n = 46). For each comparison, the relationship between genes, as well as dispersion measures of genes among case-healthy groups were calculated.

In the first data set including CRC patients *vs*. healthy subjects the correlation between the 8 genes revealed two highly related clusters of genes:

Cluster 1 genes included BAD, BAMBI, and CHD2, and Cluster 2 included FKBP5, SASH3, and NEK6. According to these findings two mathematical features were generated:

(a) Max_BAD_BAMBI_CHD2 which takes the maximum value from the three genes BAD, BAMBI and CHD2 and (b) Max_FKBP5_SASH3_NEK6 which takes the maximum value from the three genes FKBP5, SASH3 and NEK6. Logistic regression was used to develop a classification model for Cancer-Healthy using four elements:

(1) Max_BAD_BAMBI_CHD2; (2) Max_FKBP5_SASH3_NEK6; (3) EPAS1; and (4) KLF9. The model equation was: {Y~max_BAD_BAMBI_CHD2 + 5 x max_FKBP5_NEK6_SASH3 + 23 x EPAS1–3 x KLF9–25}.

**Fig 1:**
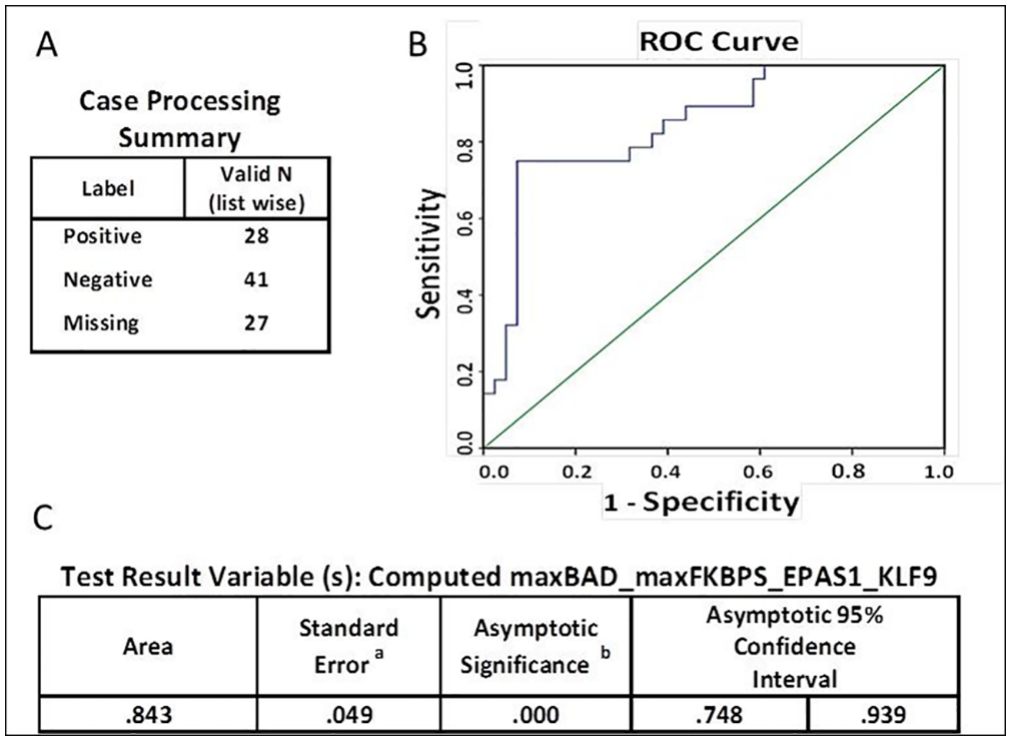
ROC analysis and AUC of cluster-model Healthy-CA. A. Case processing summary specifying valid sample numbers and labels. B. Receiver operating characteristic (ROC) curve analysis for the cluster-model Healthy-CA. C. Test Result Variable (s) of the computed Y~max_BAD_BAMBI_CHD2 + 5 x max_FKBP5_NEK6_SASH3 + 23 x EPAS1–3 x KLF9–25 model including area under the curve, standard error; asymptotic significance (and asymptotic 95% confidence interval. C.^a^. under the nonparametric assumption. C.^b^. null hypothesis: true area = 0.5.

**Fig 2:**
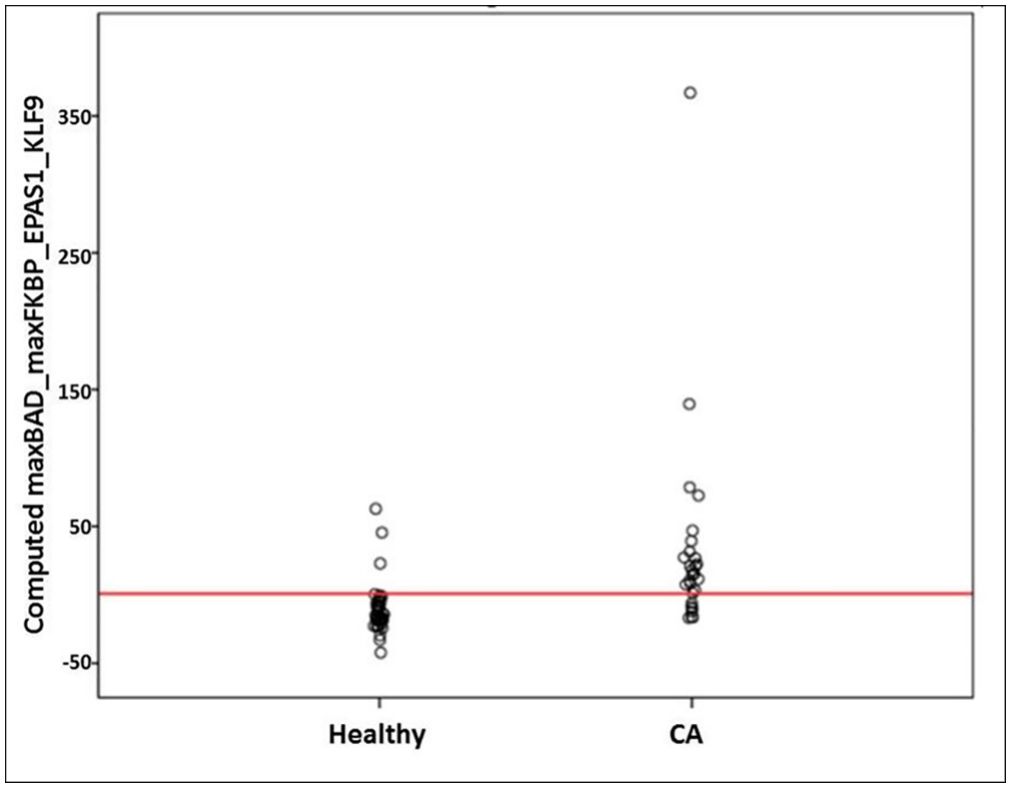
Sample distribution of cluster-model healthy controls vs. patients with colorectal carcinoma (CA). The specificity above 85% point and the maximum Youden index point meet at a point 0.84 (Red line).

**Fig 3:**
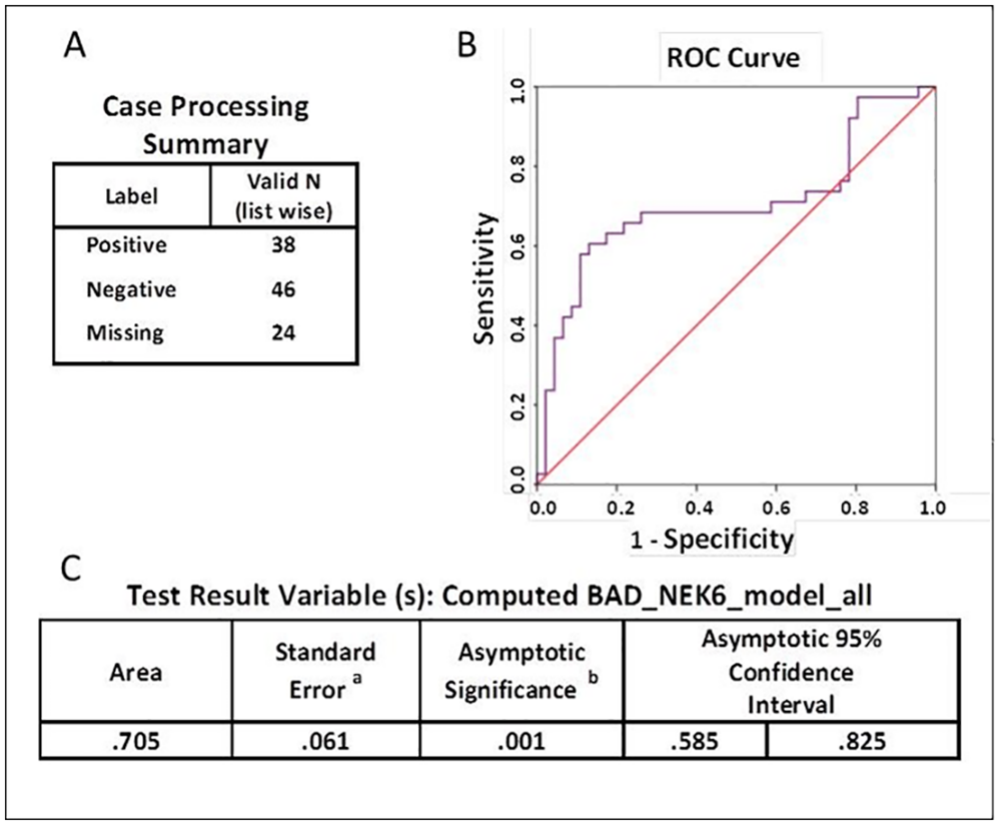
ROC analysis and AUC of cluster-model Healthy-CA. A. Case processing summary specifying valid sample numbers and labels. B. Receiver operating characteristic (ROC) curve analysis for the cluster-model Healthy-CA. C. Test Result Variable (s) of the computed Y ~ BAD+11 x NEK6-48 model all including area under the curve, standard error; asymptotic significance (and asymptotic 95% confidence interval. C.^a^. under the nonparametric assumption. C.^b^. null hypothesis: true area = 0.5.

**Fig 4:**
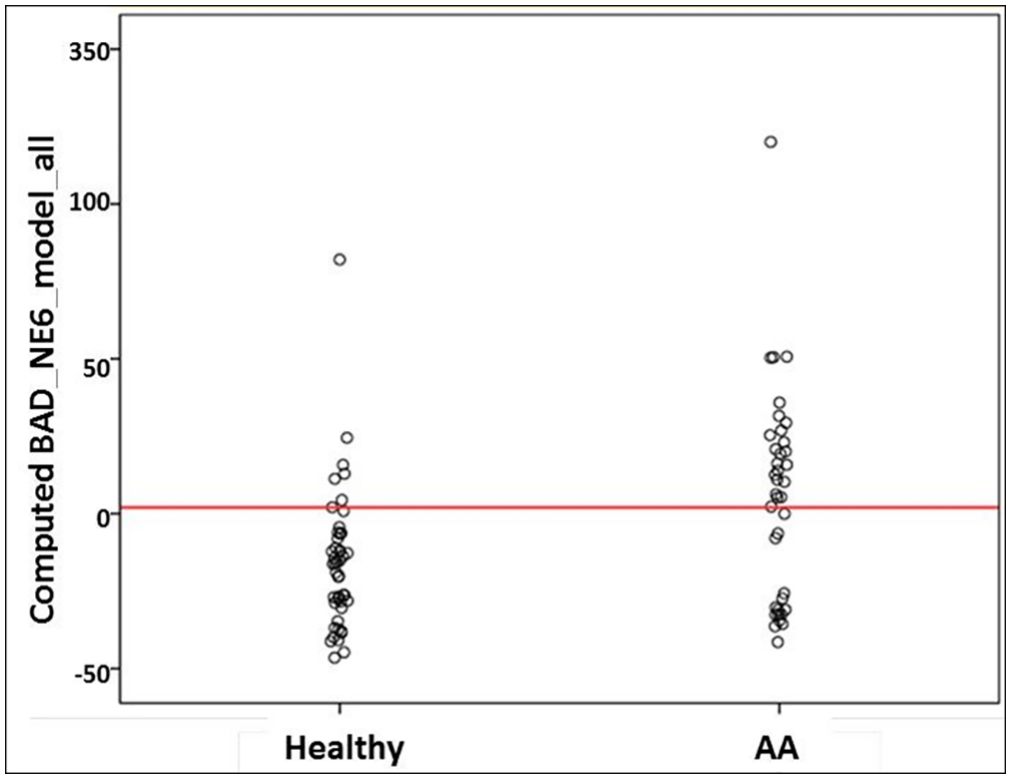
Sample distribution of cluster-model healthy controls vs. patient with advanced adenoma (AA). The specificity above 85% point and the maximum Youden index point meet at a point 2 (red line).

Receiving operating characteristic (ROC) curve analysis evaluating the discriminating capability between Cancer an Healthy subjects of the model (Fig 1) yielded 84.3% AUC (95% Asymptotic CI: 74.8%-93.9%), with P value<0.001. The specificity above 85% point and the maximum Youden index point (sensitivity + specificity -1) meet at a point 0.84 with performance sensitivity of 75% and specificity of 93% (Fig 2). For the second data set of AA patients versus healthy subjects, we used t-test and/or stepwise-regression model to select the features that participated in model building. Out of the 8 genes, BAD and NEK6 were selected and the model equation was {Y ~ BAD+11 x NEK6-48}. ROC analysis for this model yielded 70.5% AUC (95% Asymptotic CI: 58.5%-82.5%), P<0.001 for discriminating AA patients from healthy subjects (Fig 3). The specificity above 85% point and the maximum Youden index point meet at a point 2 with performance sensitivity of 60% and specificity of 87% (Fig 4).

## Discussion

Blood based tests have long been sought in the cancer detection field for their convenience and potential for higher compliance. RNA was originally thought not likely to be stable or detectable outside of the protective cellular environment. However, numerous recent studies have shown that RNA are actually stable outside of cells, [27, 28] and all species of RNA, including both coding messenger RNA (mRNA), [29] and non-coding RNA, [30, 31] can be extracted and detected in the circulating blood plasma, serum, and other bodily fluids.[32, 33] Certain fragments of RNA shed by tumors into the bloodstream can potentially be used to non-invasively screen for early-stage cancers, monitor responses to treatment and help explain why some cancers are resistant to therapies.[34]

Although the analysis of circulating free plasma RNA is a promising area, and despite all efforts to develop suitable tools for a comprehensive analysis of tumor markers from plasma RNA, the liquid biopsy is not yet routinely used as a clinical application. In this study we made a step towards a minimally-invasive, ongoing picture of cancer onset. We demonstrated that a liquid biopsy tool analyzing circulating free plasma RNA can yield a detectable genetic signature which is reproducible and repetitive using different samples.

The signature consists of a panel of 8 purified plasma RNA genes for the detection of CRC as well as AA. Our method overcame the highly degraded nature of circulating free plasma RNA by several improvements. First, we increased the efficiency of RNA purification by adding bacteriophage MS2 RNA carrier. Secondly, we targeted to amplify only short circulating free plasma fragments (about 70–100 bases) by designing appropriate primers for TaqMan^®^ reaction. And last, we improved the quantification method by calibrating the HKG primer-probe ratios to fit a low abundant RNA which is typical to circulating free plasma RNA. The method was found reliable and reproducible in repeated testing of hundreds of plasma samples.

For the CRC cohort, the ROC analysis of the selected biomarker panel generated a sensitivity of 75% and a specificity of 93% for the detection of CRC using 8-gene classification model. For the AA cohort, a sensitivity of 60% and a specificity of 87% were calculated using a 2-gene classification model.

The 8 gene panel identifying CRC patients is a unique combination of genes except for the EPAS1 gene that was also described by Mohammed N et al.[35], in plasma, and the NEK6 gene that is also one of the two genes included in the panel for detecting AA patients and was described in a recent publication by Kasap et. al.[36] The authors report the NEK6 gene as a gene with increased expression levels in correlation with the diameter of colorectal adenomatous polyps. In the present study we show that beyond polyps Nek6 is also detected in carcinoma. Except for these examples for shared genes, other signatures reported in the literature are unique discoveries.[37] Therefore, conducting extensive validation studies for such signatures is an essential step toward the future use of such panels for cancer detection. To proceed with the development of the biomarker panel a Test Set (blind testing) should be performed to validate the final biomarker composition including clinical interference cohorts such as inflammatory bowel diseases (IBD) and other solid cancers. Upon obtaining a validated signature, harmonization of the procedures is needed to provide clinical standards to introduce the liquid biopsy as a clinical detection tool in well-designed and sufficiently powered multicenter studies. There is a growing expectation that the new generation of screening tests based on molecular biomarkers present in blood should improve patient compliance in CRC screening as evidenced by the success of other screening programs such as cholesterol/lipids and prostate specific antigen (PSA).[38, 39] Our approach has a great potential as a novel and promising minimally-invasive test requiring as little as 1.6 ml plasma that can be routinely introduced to hospital labs or prescribed by general practitioners.

The choice of a blood based test such as presented here in a program of CRC screening depends on multiple features, including the potential for increased compliance, the necessary frequency of testing, and features of competing tests such as cost, which were not assessed in this study.

Ideally, a blood-based test can be a useful first line screening tool for the general population at average risk, thereby separating-out high risk and CRC patient groups. In this context it is worth to note that fecal immunochemical testing, a currently accepted strategy for noninvasive CRC screening in the average-risk population, shows a good performance for the diagnosis of CRC but overlooks almost 50% of AAs.[40] At present, it can therefore only be speculated whether a blood test with fecal occult blood test (FOBT)-like performance would produce similar reductions in incidence and mortality to those seen in previous randomized FOBT trials.

Our study design has two advantages: being representative of the general population with normal colonoscopy as well as those diagnosed with AA; demonstration of the feasibility of collection and analysis of sequential blood tests during the screening procedure. A clinically applicable screening test for CRC would also be expected to detect high risk-precancerous lesions (AA), and our panel achieved a relatively high sensitivity as well as good specificity for differentiating AA. The major limitation of our study is that it does not include an independent validation cohort.

In summary, in this study we have identified a panel of 8 plasma RNA markers as a preliminary panel for CRC detection and a subset suitable for AA detection. We suggest that this panel represents a potential strategy for noninvasive early diagnosis, as a first-line screening test for asymptomatic, average-risk population before colonoscopy. Nevertheless, this strategy needs to be independently validated in larger cohorts of patients, especially those with AAs, to assess the accuracy and potential applicability in a screening setting. In 2014 the first stool-based colorectal screening test that detects the presence of red blood cells and DNA mutations was approved owing to the detection of 42% of AAs in the average risk population (while FIT screening test detected only 24% of AAs). The Centers for Medicare & Medicaid Services (CMS) covers the new molecular test once every three years for asymptomatic average-risk patients. A molecular liquid biopsy approach should allow a low-cost, user friendly blood assay attainable to diagnostic laboratories, allowing for increasing patient compliance.

## Supporting Information

S1 File **Fig A**: **Electropherogram of circulating free plasma RNA generated by Agilent bioanalyzer chip for two RNA samples. Fig B: Expression profile of the reference genes ACTB; beta-GUS; HPRT; RPLPO; TBP; TFRC**. 13 plasma samples from 4 subject groups Healthy; Advanced Adenomas; Colorectal Cancer and IBS. The gene stability measure was calculated by DataAssist v3.01 software (ABI), and is shown beneath the plot. **Fig C: Example of calibration primer-probe ratio for TFRC gene RNA detection in plasma**.
(DOC)
