# Supplementary material for: Feasibility of Unbiased RNA Profiling of Colorectal Tumors: A Proof of Principle
Source: PLoS One. 2016 Jul 21;11(7):e0159522. doi: 10.1371/journal.pone.0159522 (PMC4956030; doi:10.1371/journal.pone.0159522)
Supplement: S1 File — Fig A: Electropherogram of circulating free plasma RNA generated by Agilent bioanalyzer chip for two RNA samples. Fig B: Expression profile of the reference genes ACTB; beta-GUS; HPRT; RPLPO; TBP; TFRC. 13 plasma samples from 4 subject groups Healthy; Advanced Adenomas; Colorectal Cancer and IBS. The gene stability measure was calculated by DataAssist v3.01 software (ABI), and is shown beneath the plot. Fig C: Example of calibration primer-probe ratio for TFRC gene RNA detection in plasma. (DOC) [file pone.0159522.s001.doc]

**S1 FileS1 File.Table A: : Gene name and primer and probe sequences for 17 gene list. The final selected 8 genes are highlighted in gray.**

| **Gene Name** | **Primer Sequences** |
| --- | --- |
| **ANXA11** | |
| Probe | TGG CCG TGG TGA AAT GTC TCA AGA |
| Primer 1 (Fw) | GGC CTT GTT GAG CCT CTC |
| Primer 2 (Rev) | GTT TTC AAT GAG TAC CAG AGA ATG AC |
| **ARHGAP15** | |
| Probe | CAG ATT GCC AAA GGA TTC AAG TTG TCC A |
| Primer 1 (Fw) | TTC AGT GCT AGA GGA TCT TTG G |
| Primer 2 (Rev) | AAT GAG TTC CTT CTA CAG TCA GAT |
| **BAD** | |
| Probe | CTG GAG CTT TGC CGC ATC TGC |
| Primer 1 (Fw) | AGG ATG AGT GAC GAG TTT GTG |
| Primer 2 (Rev) | CTG CCC AAG TTC CGA TCC |
| **BAMBI** | |
| Probe | TTC GAT GCT ACT GTG ATG CTG CCC |
| Primer 1 (Fw) | CCG TGC TGC TCA CCA AA |
| Primer 2 (Rev) | GCT CAG ATT TAC ACA TAT AAC CAG TG |
| **CCR7** | |
| Probe | TG ACC TCA TC TTG ACA CAG GCA TAC C |
| Primer 1 (Fw) | TTA AAG TTC CGC ACG TCC TT |
| Primer 2 (Rev) | TGG CTC TCC TTG TCA TTT TCC |
| **CHD2** |  |
| Probe | CGA AAT CAA ACA ATG GTT AGG GAA AGT TTC TCC |
| Primer 1 (Fw) | CCT TAC AGC AAC AGA AAG TGA AG |
| Primer 2 (Rev) | CTG AAG CCA GCT CCT GTT |
| **CHPT1** |  |
| Probe | AGC AAG TGT TCG GAA GTC TCT TCA ATC C |
| Primer 1 (Fw) | TCT CCT TCC ATG AAA CAG CAG |
| Primer 2 (Rev) | GTC ATC AAG CAC CTG AAC AG |
| **COX11** |  |
| Probe | AAA ACG CCA GTG CAGTCT CTC CT |
| Primer 1 (Fw) | CCA GTG GAA CTT TAG ACCTCA G |
| Primer 2 (Rev) | AAA TACTGT CCA GCT TCA AAT GG |
| **EPAS1** |  |
| Probe | AGA GTC ACC AGA ACT TGT GCA CCA A |
| Primer 1 (Fw) | AGC CTA TGA ATT CTA CCA TGC G |
| Primer 2 (Rev) | CTT TGC GAG CAT CCG GTA |
| **FKBP5** |  |
| Probe | TC AAA CAT CC TTC CAC CAC AGC GG |
| Primer 1 (Fw) | CAC AGT GAA TGC CAC ATC TCT |
| Primer 2 (Rev) | TGA AGA TGG AGG CAT TAT CCG |
| **HNRNPH3** |  |
| Probe | TTC AGG TTT TCA TGG TGG TCA TTT CG |
| Primer 1 (Fw) | GGA AGA GGT ATG GGA GGA CA |
| Primer 2 (Rev) | CGT ATT GGA TTT AGT GGT GAG AAG |
| **KIAA0101** |  |
| Probe | AAA CGG GGT TCC CTC CTG CAT ATT |
| Primer 1 (Fw) | TCT GCC ACT AAT TCG ACA TCA G |
| Primer 2 (Rev) | CTC CAA TTC CTT TTT GCC ACT T |
| **KIAA1199** |  |
| Probe | CCT CTC CAT CCA TCA TAC ATT CTC TCG CT |
| Primer 1 (Fw) | GAC CCA CCC ACA TAC ATC AG |
| Primer 2 (Rev) | CCC AAA GAG TTA TAG CCC ACA A |
| **KLF9** |  |
| Probe | AG TGC ATA CA GGT GAA CGG CCC |
| Primer 1 (Fw) | GGA GAA CTT TTT AAG GCA GTC TG |
| Primer 2 (Rev) | CTC CCA TCT CAA AGC CCA TT |
| **NEK6** |  |
| Probe | AG GAT CCA TG AGA ACG GCT ACA ACT TC |
| Primer 1 (Fw) | TGG CAC AGG GAG AAG AGA T |
| Primer 2 (Rev) | CGC CCT ACT ACA TGT CAC C |
| **S100A9** |  |
| Probe | AG CTC TTT GA ATT CCC CCT GGT TCA |
| Primer 1 (Fw) | CCT CCA TGA TGT GTT CTA TGA CC |
| Primer 2 (Rev) | CAA CAC CTT CCA CCA ATA CTC T |
| **SASH3** |  |
| Probe | AGA AGA GAT GGC AGA CAC TCT GGA GG |
| Primer 1 (Fw) | AGG CTG TAG TCT GGA GAT GTC |
| Primer 2 (Rev) | CAG GAA GAT GGG CAA GAT GA |

**S1 File.Figure A:**

**
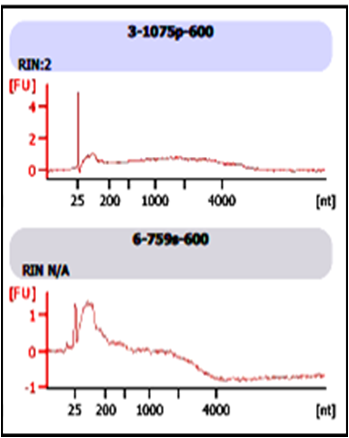
**

**S1 File. Figure** B**:**

**
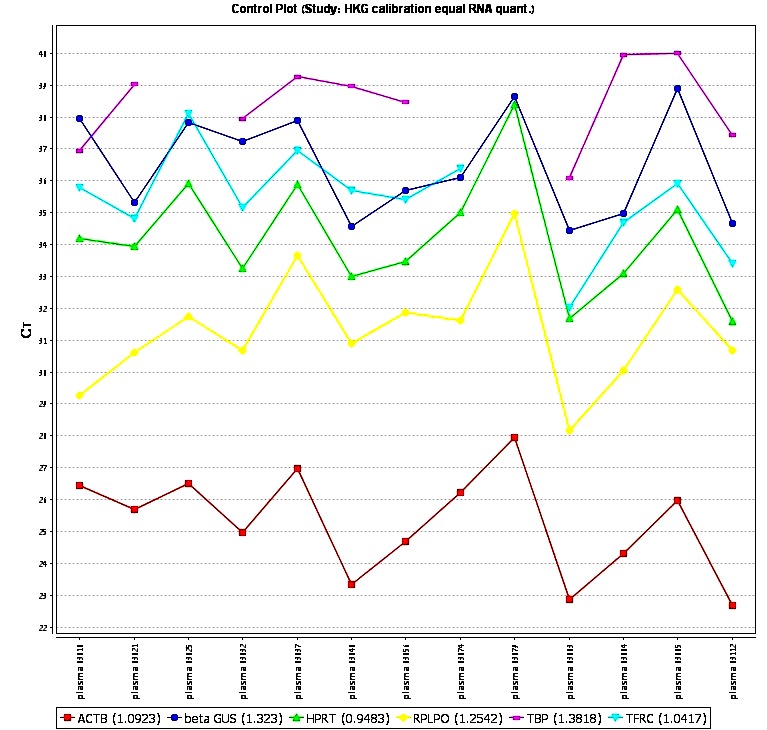
**

**S1 File.Figure C**

**
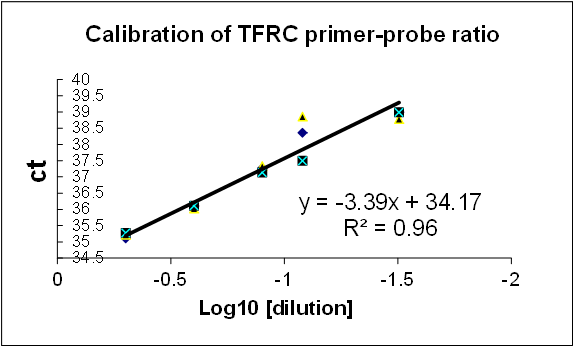
**

**S1 File Figure Legends**

**S1 File. Figure A**: **Electropherogram of circulating free plasma RNA generated by Agilent bioanalyzer chip for two RNA samples.**

**S1 File. Figure B: Expression profile of the reference genes ACTB; beta-GUS; HPRT; RPLPO; TBP; TFRC**. 13 plasma samples from 4 subject groups: Healthy; Advanced Adenomas; Colorectal Cancer and IBS. The gene stability measure was calculated by DataAssist v3.01 software (ABI), and is shown beneath the plot.

**S1 File.Figure C:** **Example of calibration primer-probe ratio for TFRC gene RNA detection in plasma**
